# Edible Environmental Enrichments in Littered Housing Systems: Do Their Effects on Integument Condition Differ Between Commercial Laying Hen Strains?

**DOI:** 10.3390/ani10122434

**Published:** 2020-12-18

**Authors:** Ruben Schreiter, Klaus Damme, Markus Freick

**Affiliations:** 1 ZAFT e.V. Centre for Applied Research and Technology, D-01069 Dresden, Germany; markus.freick@htw-dresden.de; 2 Bavarian State Farms, Research and Education Center for Poultry, D-97318 Kitzingen, Germany; klaus.damme@baysg.bayern.de; 3 Faculty of Agriculture/Environment/Chemistry, HTW Dresden—University of Applied Sciences, D-01326 Dresden, Germany

**Keywords:** egg production, animal welfare, layers, pullets, feather pecking, genotype-environment interaction

## Abstract

**Simple Summary:**

Feather pecking can occur in laying hens. It is considered a behavioral disorder and can be triggered by a variety of factors. Feather pecking leads to plumage damage, which is detrimental to the welfare and performance of the animals. The behavior of allopecking is assigned to foraging and exploration behavior. Therefore, a possible approach to reduce feather pecking is to provide manipulatable, edible objects to direct the hens’ pecking to these substrates. Previous studies could not clearly answer the question of the effects of providing such environmental enrichment materials on plumage condition and performance. In order to clarify this question and investigate possible differences between different breeding lines, 4000 pullets were kept during rearing and 2808 hens were kept during the laying period with or without additional enrichment materials (pecking stones and alfalfa bales). The results showed that the effect of enrichment materials on plumage condition and also on pecking injuries of the skin differed between genetic strains. Therefore, the recommendations for the use of enrichment materials should be revised and specified for the hybrid lines.

**Abstract:**

The aim of this study was to investigate the effect of additional enrichment materials (EMs; pecking stones and alfalfa bales) on the occurrence of plumage damage, skin injuries, and toe injuries, with an emphasis on the possible differences between commercial hybrid strains of laying hens. During rearing (weeks 1–18, 16 compartments, 4000 pullets) and laying periods (weeks 21–72, 24 compartments, 2808 hens) in a littered housing system, EMs were permanently provided to the study groups (EXP), while control groups (CON) did not receive additional EM. In a two-factorial study design (two groups with four strains) with 351 hens per variant, the brown egg-laying Lohmann Brown classic (LB) and Bovans Brown (BB) strains as well as the white egg-laying Lohmann Selected Leghorn classic (LSL) and Dekalb White (DW) strains were investigated. Compared to the CON, the EXP showed reduced body mass during rearing (*p* < 0.001) and reduced albumen consistency in the laying period (*p* < 0.001). Regarding integument condition, the LSL in the EXP showed more toe injuries than in the CON (*p* = 0.018). Remarkably, genotype-environment interactions between strains and groups were evident (*p* < 0.001). In groups with an EM supply, plumage damage decreased in LB (*p* ≤ 0.033) and LSL (*p* ≤ 0.005) but increased in BB (*p* ≤ 0.003). Moreover, there were fewer skin injuries in LSL (*p* = 0.001) but more in BB (*p* = 0.001) in groups with access to EM. In view of the diverging effects between strains, future practical recommendations for laying hen husbandry should be strain-specific.

## 1. Introduction

Feather pecking and cannibalism are behavioral disorders that can occur in laying hens in all housing systems [1]. They result in serious problems with regard to animal welfare but also in negative effects on the performance of the flocks and the economic success of laying hen farmers [2,3,4,5,6]. In feather pecking, a distinction can be made between gentle feather pecking, which does not lead to plumage loss, and severe feather pecking (SFP) [7]. SFP leads to plumage damage and increases the risk of pecking injuries to the exposed skin [8]. Pecking on the skin and underlying tissues of conspecifics is designated as cannibalism and, as with feather pecking, is not an aggressively motivated behavior [7,9].

If SFP and/or cannibalism occur in flocks, they are relevant for animal welfare, since extensive feather loss significantly restricts the well-being of the hens [5] and the pecked animals feel pain [10]. Toe pecking, which occurs especially in white egg layers, can lead to considerable pain, injuries, loss of toe limbs, and animal losses [11,12]. During this process, hens often peck their own toes until injuries occur [13]. Although beak trimming at chick age cannot prevent allopecking behaviors, it can significantly reduce plumage damage, injuries, and increased animal losses [14,15,16]. Therefore, with the abandonment of beak trimming in several European countries (e.g., Austria, Norway, Germany), the risk of integument damage and animal losses has increased [17,18]. Consequently, efforts to counteract the causes of allopecking behaviors must be intensified. Key measures for laying hen farmers include improving the adaption period after transfer to the laying stable, increasing the structuring of the housing environment, using flicker-free lighting, optimizing the feed composition, and reducing the level of harmful gases in the stable [9].

Genetics, feeding, housing, and management are regarded as central complexes in the multifactorial causal structure of SFP and cannibalism [19,20,21,22]. Since SFP is regarded as a misdirection of foraging and feed intake behavior [23,24], the supply of manipulatable materials to the birds is of high relevance [5]. There is no consistent knowledge of the effect of additional enrichment materials (EMs) on the prevalence of SFP or plumage damage in laying hens kept in littered housing systems (reviewed by [25]). With the addition of cereals to the litter during rearing, [26] observed less feather pecking in the laying period. Sand and peat during rearing as well as straw provided in baskets during the laying period could reduce plumage damage in laying hens [27]. Such an effect was achieved in [28] by enriching the rearing stable with structural elements and novel objects. In pullets, the use of strings or pecking stones and alfalfa bales could reduce the occurrence of feather pecking [6,29]. Moreover, less plumage damage was observed due to the additional supply of maize silage, pea/barley silage, and carrots [30], as well as pecking stones and alfalfa bales [31]. On the other hand, other authors did not observe the positive effects of these EMs [16,32,33,34]. However, none of these studies examined different genetic strains of white egg and brown egg-laying hens in parallel. Therefore, knowledge of possible genotype-environment interactions (GEIs) in the provision of EMs regarding SFP and cannibalism is lacking. The existence of GEIs in laying hens has been demonstrated between strains and housing systems regarding performance characteristics or feeding behavior [35,36,37,38]. Brown egg layers in floor housing showed a lower feed intake than in small-group housing, whereas no differences in feed intake between the housing types were found in white egg layers [38]. The laying performance of different hybrid strains varied between different test environments as a result of GEI [35,36,37].

The aim of this study was to examine the effect of EM on the integument condition in laying hens and to identify possible differences between strains. The following hypotheses were generated: (1) by providing EM, the prevalence of severe plumage and skin damage can be reduced in week 72 (i.e., at the end of the laying period), and (2) the effects of EM supply on integument condition differ between the strains.

## 2. Materials and Methods

The study was conducted at the Bavarian State Research Center for Agriculture in the Department of Applied Research and Education in Poultry (Kitzingen, Germany) on non-beak trimmed pullets and laying hens from January 2019 to May 2020. In a single run, the effect of additional EM on the prevalence of integumentary damage, animal development, and laying performance was investigated in different hybrid strains.

### 2.1. Housing and Management

The rearing stable was equipped with a two-floored aviary system (Natura Filia, Big Dutchman AG, Vechta-Calveslage, Germany) and divided into 16 identical compartments (12.9 m^2^ of floor area each, including 6.6 m^2^ grids and 6.3 m^2^ litter, and 19.4 hens/m^2^ usable area, equipped with perches, a flat chain feeding system and nipple drinkers). On the first day of life, 250 chicks per compartment were placed in the lower aviary level. On day 8, half of the chicks per compartment were transferred to the upper aviary level. Prior to housing, 80% of the grid area was covered with chick paper (roll corrugated board, REKA Wellpappenwerke, Kitzingen, Germany), which was removed at the end of week 5. Chick starter feed (see below) was provided on the chick paper from day 1 to 8 to encourage the chicks to take up feed. On day 35, the aviary segments were opened to allow access to the floor area, which was littered with softwood shavings (Premiumspan^®^, Hobelspanverarbeitung GmbH, Dittersdorf, Germany). To maintain an attractive, loose litter, the area was re-littered with softwood shavings in identical quantities in each compartment at two-week intervals. The lighting of the barn was provided by Light-Emitting Diode (LED) tubes (FlexLED, Big Dutchman AG, Vechta-Calveslage, Germany), which were installed in the longitudinal direction of the barn centrally above each scratching area and also in the lower level of the aviary.

In week 19, the pullets were transferred to a laying stable with 24 compartments (15.6 m^2^ of floor area each; 7.5 hens/m^2^ usable area). Each compartment housed 117 hens (hens housed, HH) and was equipped with an automatic round trough feeder (Big Dutchman AG, Vechta-Calveslage, Germany), a nipple drinker line (Big Dutchman AG, Vechta-Calveslage, Germany), a double-storey family nest with Astroturf mats (VencoTec GmbH, Holzheim, Germany, each storey 3.3 × 0.5 m) and perches (self-made from wood, rectangular cross-section 4 × 5 cm, total length 14.8 m). One third of the floor area was littered with softwood shavings (Premiumspan^®^, Hobelspanverarbeitung GmbH, Dittersdorf, Germany), while the remaining two thirds consisted of perforated flooring (plastic grids, Big Dutchman AG, Vechta-Calveslage, Germany) with an underlying manure belt (Big Dutchman AG, Vechta-Calveslage, Germany). At four-week intervals, the litter was removed from the compartments and replaced by new softwood shavings (see above). The lighting of the laying stable was provided by LED lamps (LED lamp, Big Dutchman AG, Vechta-Calveslage, Germany), which were located in each compartment above the littered area.

For demand-oriented feeding during the rearing period, a three-phase feeding program (from Deutsche Tiernahrung Cremer GmbH & Co. KG, Regensburg, Germany) with complete chick feed (weeks 1–10, Bonimal GK KAM, Deutsche Tiernahrung Cremer GmbH & Co. KG, Regensburg, Germany) and complete pullet feed (weeks 11–18, Bonimal GK JAM, Deutsche Tiernahrung Cremer GmbH & Co. KG, Regensburg, Germany) in the rearing stable and a pre-laying diet after moving to the laying stable (weeks 19–20, Bonimal GK Vorlegemehl, Regensburg, Germany) was provided. Then, birds were fed complete feeds for laying hens (from Deutsche Tiernahrung Cremer GmbH & Co. KG, Regensburg, Germany) designed for phase I (weeks 21–60, Bonimal GK LAM 40, Regensburg, Germany) or phase II (weeks 61–72, Bonimal GK LAM 44, Regensburg, Germany). The nutrient and active substance levels were based on current recommendations [39]. Feed and drinking water were available ad libitum to the animals during the rearing and laying periods. All the feeds of the study had a mashed structure.

A regulated step-down–step-up light program, based on the current management recommendations [39], was used for the targeted control of animal development and laying maturity. The window surfaces in the rearing and laying stables were completely darkened. The average lighting intensity at animal height in the feeding and littered area was 35 lux in weeks 1–3 and 20 lux from week 4 onwards. In order to ensure stable animal health and to reduce animal losses, a vaccination program appropriate for the stock, region, and intended use was undertaken following the recommendations of [39].

### 2.2. Animals, Study Design, and Data Collection

The animals were kept in accordance with the legal measures of the EU (Council Directive 1999/74/EC, minimum standards for the protection of laying hens) [40] and Germany (Animal Welfare Act; Animal Protection Keeping of Production Animals Order) [41,42]. In particular, the principles and specific guidelines of the Guide for the Care and Use of Agricultural Animals in Research and Teaching [43] were implemented. In accordance with Directive 2010/63/EU of the European Parliament and of the Council on the protection of animals used for scientific purposes [44], no invasive treatment of the hens was performed.

**Figure 1:**
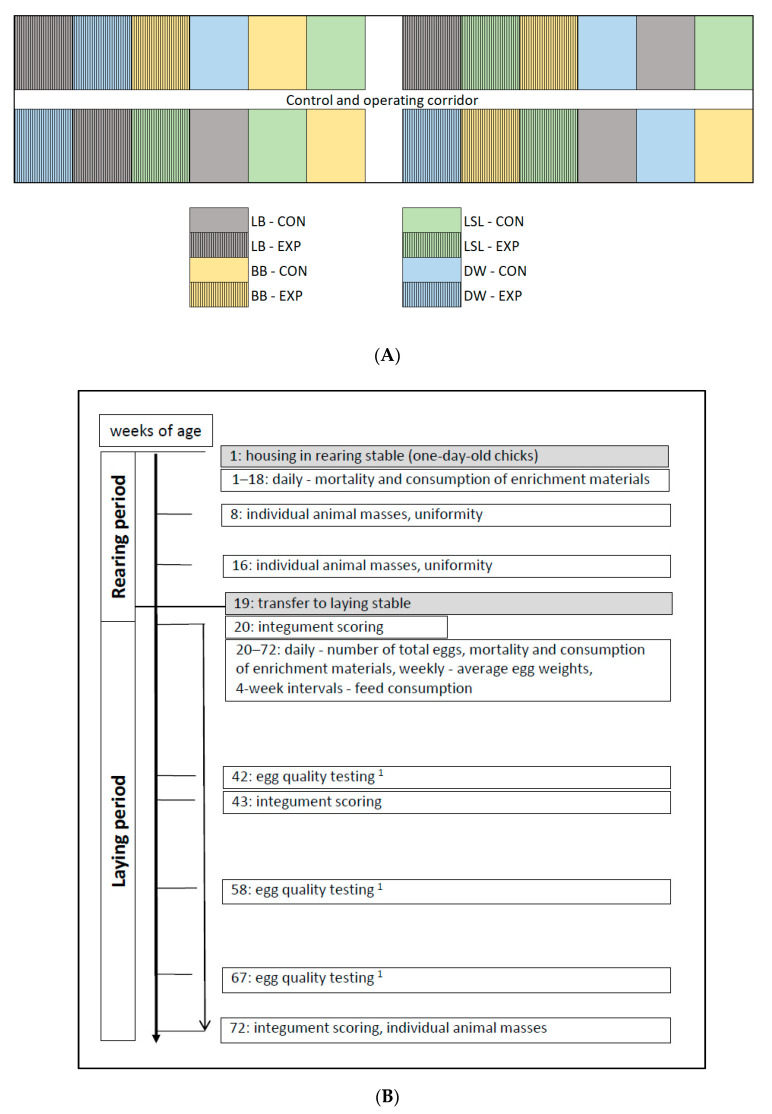
Study design: (**A**) Assignment of the four hybrid strains and two study groups to the compartments in the laying stable. (**B**) Times of scorings and data collection. Grey fields represent housing events. LB (CON—control group)—Lohmann Brown classic without supply of enrichment materials; LB (EXP—experimental group)—Lohmann Brown classic with supply of enrichment materials; BB (CON)—Bovans Brown without supply of enrichment materials; BB (EXP)—Bovans Brown with supply of enrichment materials; LSL (CON)—Lohmann Selected Leghorn classic without supply of enrichment materials; LSL (EXP)—Lohmann Selected Leghorn classic with supply of enrichment materials; DW (CON)—Dekalb White without supply of enrichment materials; DW (EXP)—Dekalb White with supply of enrichment materials. ^1^—individual egg weights, breaking strength of the eggshells, and albumen consistency.

For this study, 1000 one-day-old chicks each of the commercial brown egg-layer hybrid strains Lohmann Brown classic (LB; Lohmann Tierzucht, Cuxhaven, Germany; purchased from LSL Rhein Main hatchery, Dieburg, Germany) and Bovans Brown (BB; Hendrix Genetics, Boxmeer, The Netherlands; purchased from Het Anker BV hatchery, Ochten, The Netherlands) as well as 1000 one-day-old chicks each of the commercial white egg-layer hybrid strains Lohmann Selected Leghorn classic (LSL; Lohmann Tierzucht, Cuxhaven, Germany; purchased from LSL Rhein Main hatchery, Dieburg, Germany) and Dekalb White (DW; Hendrix Genetics, Boxmeer, The Netherlands; purchased from Het Anker BV hatchery, Ochten, The Netherlands) were housed in the rearing stable with 250 chicks per compartment. In week 19, the hens were transferred to a laying stable. Only hens from the same rearing compartment were housed in one laying compartment. For each of the four strains under investigation, 702 hens were placed in the 24 compartments (i.e., 117 hens per compartment). The assignment to the compartments was alternated for the four hybrid strains and blocked for the two study groups (see below) (Figure 1A). For each variant (i.e., four strains and two groups resulting in eight variants), two compartments were available for rearing and three for the laying period.

Regarding the supply of edible enrichment materials in addition to the litter, birds of each strain were divided into two different groups. In the control group (CON), no EM was supplied during the rearing and laying periods apart from the chick paper and litter. In the experimental group (EXP), additional EMs were available to the birds from the first day of life. For that purpose, pecking stones (Vilolith^®^ medium, Deutsche Vilomix Tierernährung GmbH, Neuenkirchen-Vörden, Germany) and hard-pressed alfalfa bales (Einstreuprofi, Seelingstädt, Germany) were supplied. The EMs were provided ad libitum and were replaced in the compartment shortly before complete consumption. Pecking stones and alflafa bales were placed centrally in the aviary compartment (weeks 1–5) and in the littered area (from week 6) next to each other. From week 8 onwards, EMs were offered in the littered area only. In the phase with closed aviary segments (weeks 1–5), pecking stones and alfalfa blocks were placed in flat feed trays (Futterteller, Siepmann GmbH, Herdecke, Germany). Two pecking stones and four alfalfa bales were permanently available to the animals in each compartment.

In this observational longitudinal study, the characteristics of integument condition and body mass, laying performance, egg quality, mortality, feed consumption, and EM consumption were assessed (Figure 1B).

For the indirect determination of the occurrence of SFP and cannibalism, a scoring of the integument was performed in weeks 20, 43, and 72. These dates were chosen to depict the most important stages of the laying period regarding plumage condition—i.e., laying maturity (week 20), peak of egg mass production (week 43) and the final of the laying period (week 72). For integument scoring, individual birds were regarded as experimental units, and the required sample size was calculated from our preliminary investigations (data not shown) using a web-based tool [45]. In order to prove differences of 20% in the proportion of birds with integument damages with a statistical power of 0.80 and a significance level set at α = 0.05, a sample size of a minimum of 72 birds per variant was necessary. At each observation date, a subset of 25 randomly selected hens per compartment (i.e., *n* = 75 per variant) was therefore scored. The scoring was based on the Welfare Quality^®^ protocol [46] modified by [47] for the integument traits of plumage damage, skin injuries, toe injuries, and foot pad condition. For plumage scoring, the following scores were used: 0 = intact plumage with featherless areas up to 1 cm; 1 = slight plumage damage with featherless areas >1 to 5 cm; 2 = severe plumage damage with featherless areas >5 cm. Skin lesions were classified by score 0 = intact skin; score 1 = small pecking lesions up to 1 cm in diameter; score 2 = lesions and/or wounds larger than 1 cm at the largest diameter.

The scoring of the plumage was differentiated according to the back, belly (including cloacal region and ventral rump) and dorsal neck. In addition to the three individual scores, a total plumage score was calculated on the individual animal level by adding the individual scores. The feathers of the front of the neck and the breast were not included in the scoring, as feather damage in these areas due to mechanical stress from the feeding trough does not provide strong evidence for SFP [8]. For the assessment of skin injuries, all the feathered body regions were relevant. For the evaluation of toe injuries, the toes with the most severe damage were decisive for the assigned score. Integument scoring was always performed by the same observer who had previously completed training in the application of this scoring system with 3500 hens. The investigator was blinded for the study group.

During the rearing period (weeks 1–18), the feed consumption was determined in each compartment at the end of the 14-day period by recording the quantity consumed using a feeding computer (Fancom 743, Fancom, Panningen, The Netherlands) and a manual back weighing (Defender 3000 scale). Feed consumption was determined by continuous feed and back weighing (Defender 3000 scale, Ohaus, Parsippany, NJ, USA) in four-week intervals during the laying period. Each pecking stone and alfalfa bale of each compartment was weighed during rearing and the laying period (DE6 K2N scale, Kern, Balingen, Germany). Animal losses were recorded daily. Dead hens with bloody skin injuries (diameter ≥1 cm) or skin injuries and bloody surrounding plumage were classified as mortality due to skin cannibalism, and those with toe injuries as mortality due to toe cannibalism. In weeks 8 and 16, the individual animal masses were determined using the Flexscale (Big Dutchman AG, Vechta-Calveslage, Germany) by weighing 50 animals per compartment (two compartments per variant, *n* = 100 animals). In week 72, all remaining hens were weighed (*n* = 2621). At the same time, the uniformity for each compartment was calculated on the basis of these individual animal masses. The uniformity indicates the proportion of animals weighed in a sample with respect to a body mass that lies within ±10% of the arithmetic mean of the sample [48] During the laying period, the number of total eggs for each compartment was recorded daily. The average egg weight was determined weekly by weighing with a digital scale (Kern DE6 K2N, Kern, Balingen, Germany) all individual eggs of one daily clutch of each compartment. The laying performance per average hen and per hen housed was calculated according to [49]. Egg quality traits were assessed in weeks 42, 58, and 67 in 15 randomly selected eggs per compartment in the same way as reported by [49] for the egg quality assessments in laying hen performance tests. The egg weight (Navigator NV1101 digital scale, Ohaus, Parsippany, NJ, USA), breaking strength of the egg shells (FEST V2.0 egg shell tester, Futura, Lohne, Germany), and albumen height (1/A 2001 albumen altimeter, Futura, Lohne, Germany) were measured. Based on the egg weight and albumen height, the Haugh units (HU) for characterizing albumen consistency were calculated [50].

### 2.3. Statistical Analyses

Microsoft Excel^®^ (Version 2013, Microsoft Corporation, Redmond, WA, USA) was used for the data collection and processing and the creation of selected diagrams. In the figures illustrating the plumage condition (see the Results section), the presented proportion per score corresponds to the arithmetic mean of the three scored plumage regions. For further descriptive and inferential statistical analyses, the Standard SAS program package (Version 9.4., SAS Institute Inc., Cary, NC, USA) and the IBM SPSS Statistics program (Version 23, SPSS Inc., Chicago, IL, USA) were used. The normal distribution of the residuals was tested using the Kolmogorov–Smirnov test [51].

For normally distributed data (i.e., egg number per hen housed, egg number per average hen, egg mass per hen housed, egg mass per average hen, egg weight, uniformity, feed, and EM consumption and feed conversion), two-factorial ANOVA linear models were calculated [52] with the fixed effects of strains, groups and the strain–group interaction. For post-hoc pairwise comparisons, the GT2 (Generalized Tukey 2) test according to Hochberg was used [51,53]. To control for the false discovery rate due to multiple testing, the Benjamini–Hochberg procedure was employed [54]. For the analysis of individual animal body masses during the rearing period and egg quality traits in the laying period (albumen consistency and breaking strength), ANOVA linear models with groups and strains as between-subject effects and age as a within-subject effect was used, due to repeated measurements [53].

To evaluate the effects of the fixed factors of strains, hybrid types (i.e., white egg layers vs. brown egg layers), study groups, and their interactions on the ordinally scaled integument characteristics (i.e., total plumage score, skin injuries, and toe injuries) as well as on the metrically scaled traits without normal distribution (cumulative mortality, mortality due to skin or toe cannibalism), non-parametric tests were used. For differences between the strains, the Kruskal–Wallis test was applied. As a test for differences between the enrichment groups or in the presence of significant differences between the strains, a pairwise comparison was performed using the Mann–Whitney U test [52]. A correction for multiple testing was not implemented, since—in particular with regard to the integument traits—the study was considered as an explorative approach and the univariate analyses were performed in preparation for the regression models, as described below [54].

Multiple logistic regression models with total plumage score and skin injuries (i.e., the main outcomes of the study) as dependent variables and strains, groups, ages, compartment, and strain–group interactions as independent variables were fitted to the data using binary logistic regression (BLR) models [55]. All the independent variables were forced into the models (with the inclusion method). Multiple logistic rather than ordinal regression models were used because some scores were occupied by only very few observations. For multiple logistic regressions, the ordinal data scaling (as defined by [46,47]) was transformed into a nominal scaling (the total plumage score was 0 for scores of 0 and 1 and 1 for a score of ≥2; the skin injury score was 0 for a score of 0 and 1 for a score of ≥1). The absence of multicollinearity was ensured by the calculation of Pearson’s correlation coefficient and by a collinearity diagnosis with a variance inflation factor and condition index [56,57]. Nagelkerke’s R^2^ values, which give an indication of the extent of the variation of the dependent variables explained by the model, were calculated. Nagelkerke’s R^2^ values ≥ 0.5 were considered as very good and values in the range 0.4 ≤ R^2^ < 0.5 as good [58]. In all of the described inferential statistical analyses, differences were considered statistically significant for *p* ≤ 0.05 and tended to be significant if 0.05 < *p* ≤ 0.1.

## 3. Results

### 3.1. Integument Condition

The univariate analyses revealed strain effects on plumage condition on all scoring dates (i.e., weeks 20, 43 and 72; *p* < 0.001) for the occurrence of skin injuries in weeks 43 (*p* < 0.001) and 72 (*p* = 0.005), and toe injuries in weeks 43 (*p* < 0.001) and 72 (*p* < 0.001) (data not shown). Significant effects of the hybrid type on the plumage condition could be demonstrated in weeks 20, 43 and 72 (*p* < 0.001 each) with less plumage damage in the white egg layers (week 20: 98.6% score 0, 1.3% score 1 and 0.1% score 2; week 43: 76.6% score 0, 13.7% score 1 and 9.8% score 2; week 72: 19.8% score 0, 42.6% score 1 and 37.7% score 2) in comparison to the brown egg layers (week 20: 96.0% score 0, 3.4% score 1 and 0.6% score 2; week 43: 55.7% score 0, 23.9% score 1 and 20.4% score 2; week 72: 13.3% score 0, 35.2% score 1 and 51.5% score 2). An effect of the hybrid type regarding the skin lesions was found in week 72 (*p* < 0.001; brown egg layers: 66.0% score 0, 22.0% score 1 and 12.0% score 2; white egg layers: 77.0% score 0, 21.7% score 1 and 1.3% score 2), but not in weeks 20 (*p* = 1.000) and 43 (*p* = 0.602). With the exception of 0.3% moderate toe injuries in week 43, all of the brown egg-layer hens had intact toes, while the white egg layers showed significantly more toe injuries in weeks 43 (*p* < 0.001; 91.7% score 0, 5.3% score 1 and 3.0% score 2) and 72 (*p* < 0.001; 91.0% score 0, 0.7% score 1 and 8.3% score 2).

**Figure 2:**
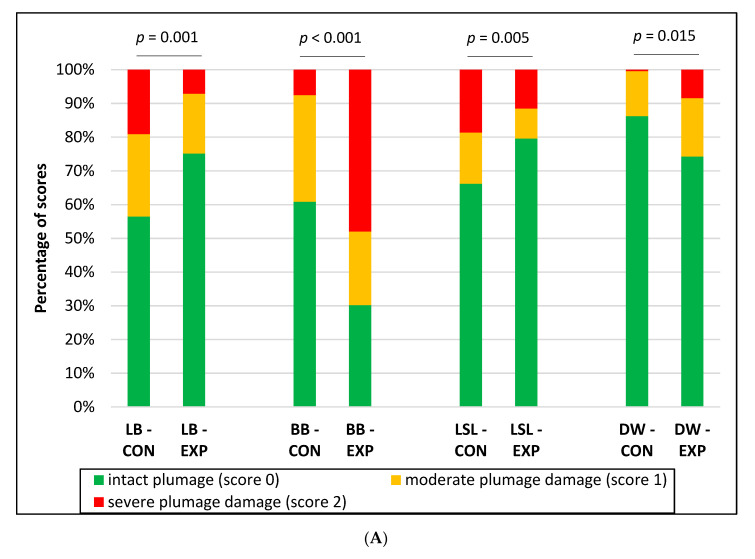

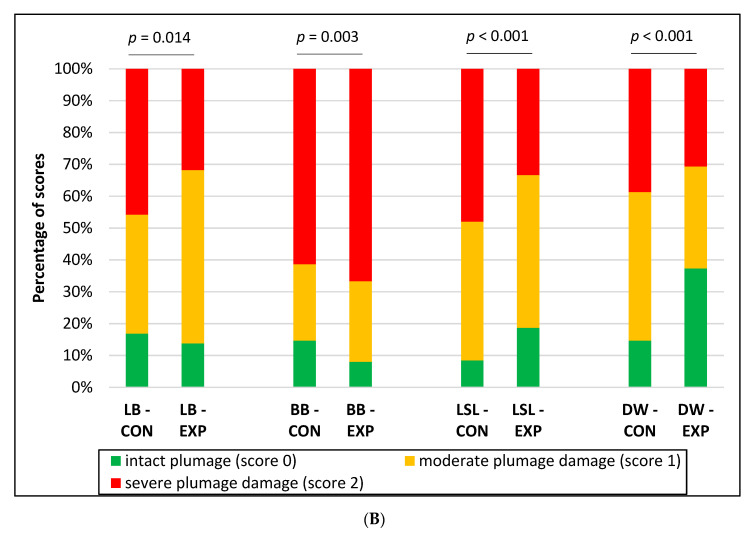
Effect of strain and supply of edible enrichment materials on plumage condition in weeks 43 (**A**) and 72 (**B**). Presented *p*-values refer to the analysis of the total plumage score. LB (CON)—Lohmann Brown classic without supply of enrichment materials; LB (EXP)—Lohmann Brown classic with supply of enrichment materials; BB (CON)—Bovans Brown without supply of enrichment materials; BB (EXP)—Bovans Brown with supply of enrichment materials; LSL (CON)—Lohmann Selected Leghorn classic without supply of enrichment materials; LSL (EXP)—Lohmann Selected Leghorn classic with supply of enrichment materials; DW (CON)—Dekalb White without supply of enrichment materials; DW (EXP)—Dekalb White with supply of enrichment materials.

**Figure 3:**
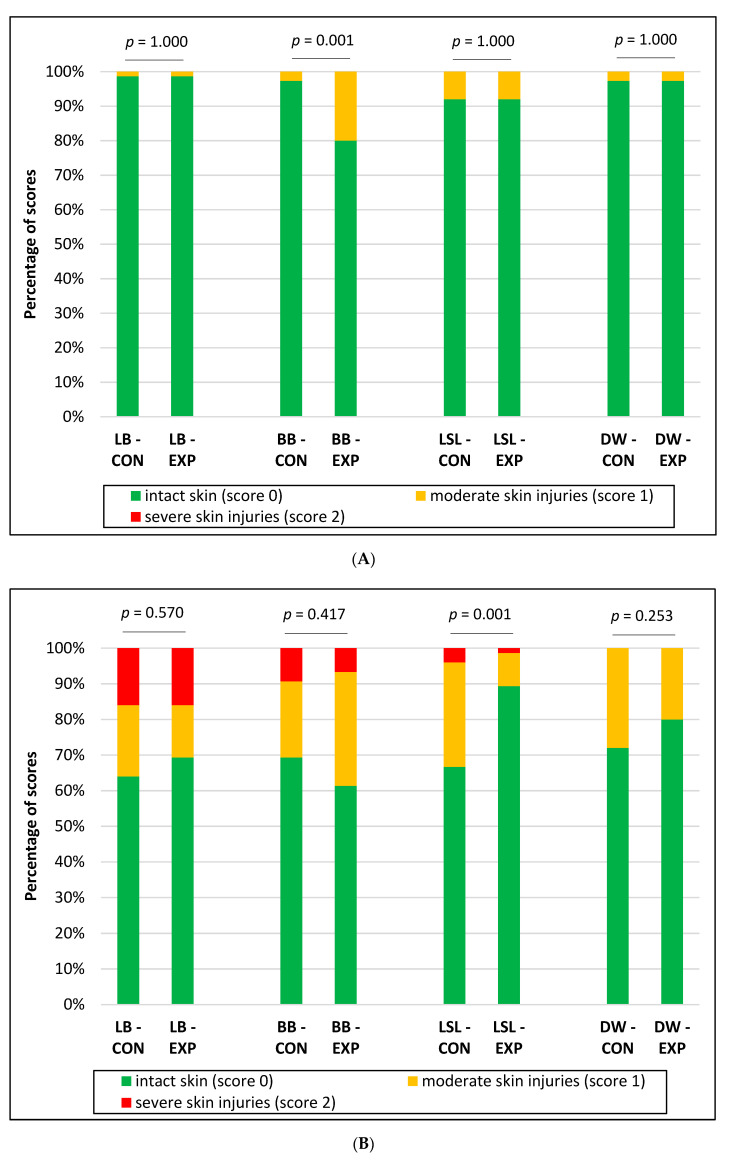
Effect of strain and supply of edible enrichment materials on skin condition in weeks 43 (**A**) and 72 (**B**). LB (CON)—Lohmann Brown classic without supply of enrichment materials; LB (EXP)—Lohmann Brown classic with supply of enrichment materials; BB (CON)—Bovans Brown without supply of enrichment materials; BB (EXP)—Bovans Brown with supply of enrichment materials; LSL (CON)—Lohmann Selected Leghorn classic without supply of enrichment materials; LSL (EXP)—Lohmann Selected Leghorn classic with supply of enrichment materials; DW (CON)—Dekalb White without supply of enrichment materials; DW (EXP)—Dekalb White with supply of enrichment materials.

Hens of all strains and groups already showed the first plumage damages—to a small extent—in week 20. At that time, the plumage loss in LB hens in the EXP groups was lower than in the CON (CON: 91.6% score 0, 7.1% score 1, 1.3% score 2; EXP: 94.7% score 0, 4.0% score 1, 1.3% score 2) (*p* = 0.033), while it tended to be higher in BB in the EXP (CON: 100.0% score 0; EXP: 96.0% score 0, 4.0% score 1) (*p* = 0.087). No differences in plumage condition between the CON and EXP could be identified for LSL (*p* = 0.992) and DW (*p* = 0.188). Diverging strain effects were also evident in week 43 (Figure 2A). The supply of EM reduced plumage damage in LB and LSL but increased plumage loss in BB and DW. With the exception of DW, these effects were confirmed in week 72 (Figure 2B). At that time, plumage damage in DW was more pronounced in CON. All the birds showed an intact skin in week 20. However, first moderate injuries were observed in week 43 in all strains and groups, while severe skin injuries occurred in week 72 in LB (CON and EXP: 16.0% each), BB (CON: 9.3%, EXP: 6.7%) and LSL (CON: 4.0%, EXP: 1.3%). Within the strains, the prevalence of skin lesions was lower in BB in the CON groups in week 43 and in LSL in the EXP groups in week 72 (Figure 3).

Toe injuries occurred exclusively in LSL hens in weeks 43 and 72. The prevalence within that strain was higher in the EXP compared to the CON in weeks 43 (CON: 90.7% score 0, 5.3% score 1, 4.0% score 2; EXP: 76.0% score 0, 16.0% score 1, 8.0% score 2) (*p* = 0.018) and 72 (CON: 89.4% score 0, 1.3% score 1, 9.3% score 2; EXP: 74.7% score 0, 1.3% score 1, 24.0% score 2) (*p* = 0.018).

Given the partly divergent effects of EM between strains in the univariate analyses, in addition to the effects of strains, groups, and ages on plumage condition and skin lesions, possible strain–group interactions were analyzed in the BLR models (Table 1). With respect to plumage condition, the effects of strains, EM groups, ages, and compartments were observed. Nagelkerke’s R^2^ values showed a very good explanatory quality of the final model for this trait (Nagelkerke’s R^2^ = 0.726). An age effect was observed in skin lesions (Nagelkerke’s R^2^ = 0.326). The significant strain–group interactions in both plumage conditions and skin injury models were highlighted. Compared to LB–CON as a basis, the effect of EM provision on plumage condition was different in the BB and DW. In terms of skin lesions, only the BB hens differed from the previously mentioned base (Table 1).

### 3.2. Body Mass, Performance Traits, EM Consumption and Egg Quality

**Figure 4:**
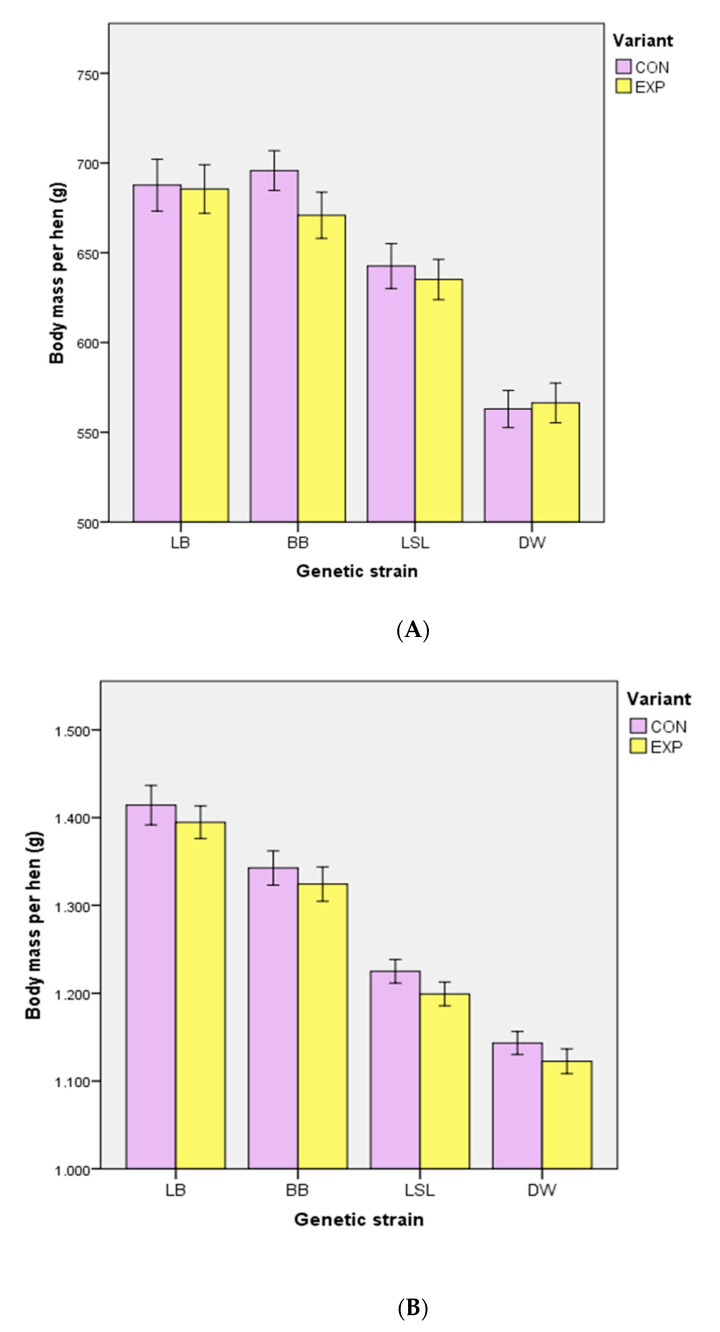
Effect of edible enrichment materials on body mass in different layer strains during the rearing period in weeks 8 (**A**) and 16 (**B**). In a repeated-measures ANOVA model, lower body masses were found in BB (*p* = 0.003) and LSL (*p* = 0.002) within EXP compared to CON, but not in LB (*p* = 0.138) and DW (*p* = 0.233). CON—control group (without additional enrichment materials); EXP—experimental group (supply of additional enrichment materials); LB = Lohmann Brown classic; BB = Bovans Brown; LSL = Lohmann Selected Leghorn classic; DW = Dekalb White. Error bars represent standard deviation.

During rearing, body mass decreased with the supply of EM (*p* < 0.001) when evaluated over all strains (data not shown). Within the strains, this effect was present for BB (weeks 8 and 16: *p* = 0.003) and LSL (week 8 and 16: *p* = 0.002), but not for LB (weeks 8 and 16: *p* = 0.138) and DW (week 8 and 16: *p* = 0.233) (Figure 4). A significant interaction between strains and groups did not exist (*p* = 0.511). Uniformity in weeks 8 (CON: 72.8 ± 7.4%, EXP: 72.8 ± 6.1%) and 16 (CON: 86.5 ± 6.9%, EXP: 88.3 ± 4.7%) was not affected by enrichment (*p* ≥ 0.424). Cumulative mortality was 1.6% (median, minimum–maximum: 0.8–4.4%) in CON and 1.0% (0.4–3.6%) in EXP during rearing (*p* = 0.195), with no cannibalism-related animal losses. The consumption of pecking stones varied between the strains, with a higher consumption in BB hens (51.1 ± 3.6 g) than in LB (40.3 ± 2.5 g), LSL (41.7 ± 3.5 g) and DW (37.4 ± 3.2 g) (*p* = 0.048). During rearing, the hens consumed 29.9 ± 2.8 g of alfalfa material, with no differences between the strains (*p* = 0.101).

In the laying period (52 weeks), average hens laid 324.3 ± 14.9 eggs, each with an average mass of 62.7 ± 1.7 g, and required 2.225 ± 0.110 kg of feed to produce 1 kg of egg mass (mean ± SD). These performance characteristics differed between the strains (*p* < 0.001), but not between the EXP and CON (*p* ≥ 0.582).

The daily feed consumption per hen was not affected by the EM group (CON: 123.3 ± 2.6 g; EXP: 124.5 ± 3.7 g) (*p* = 0.246). In week 72, body mass differed between the strains (*p* < 0.001; LB: 1925 ± 141 g, BB: 1912 ± 134 g, LSL 1767 ± 118 g, DW: 1724 ± 109 g), but not between the enrichment groups (*p* = 0.963; CON: 1832 ± 177 g, EXP: 1834 ± 181 g).

The consumption of alfalfa bales per hen varied between the strains (*p* = 0.001; mean ± SD, LB: 256.5 ± 39.3 g, BB: 209.5 ± 21.9 g, LSL: 131.0 ± 16.5 g, DW: 131.8 ± 16.5 g), but the pecking stone consumption did not (over all strains: 629 ± 112.7 g; *p* = 0.353). Cumulative mortality during the laying period was 5.5% over all strains (median, minimum–maximum: 0.0–24.0%), with a lower mortality in DW at 1.8% (1.6–5.6%) compared to the other strains, with 6.0% for LB (0.0–24.0%), 4.1% for BB (1.6–9.6%) and 8.5% for LSL (5.5–17.6%) (*p* = 0.039). EM supply had no effect on cumulative mortality (CON: 5.5%, 1.6–24.0%; EXP: 6.4%, 0.0–17.6%) (*p* = 0.932) and losses due to skin cannibalism (CON: 0.4%, 0.0–16.8%; EXP: 0.4%, 0.0–6.4%) (*p* = 0.977). Within the strains, cannibalism losses were numerically higher in the CON for LB (CON: 3.6%, 1.6–16.8%; EXP: 0.0%, 0.0–4.8%) (*p* = 0.268), but lower in the CON for BB (CON: 0.8%, 0.0–0.9%; EXP: 6.3%, 0.0–6.4%) (*p* = 0.376). Animal losses due to toe cannibalism only occurred in LSL (*p* < 0.001). Without a significant effect of EM group, losses due to toe damage were numerically lower in the CON at 0.9% (0.9–6.4%) than in the EXP at 3.8% (1.8–12.0%) (*p* = 0.268).

**Figure 5:**
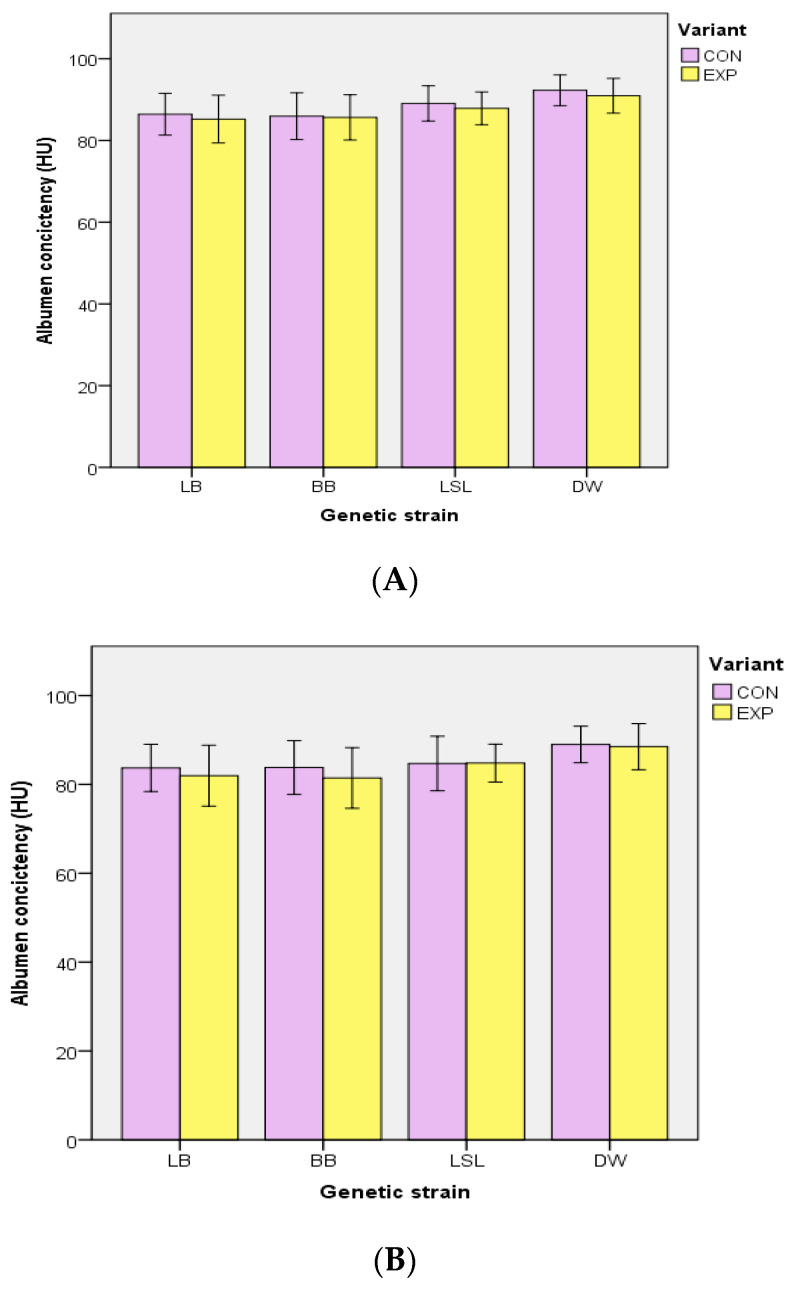

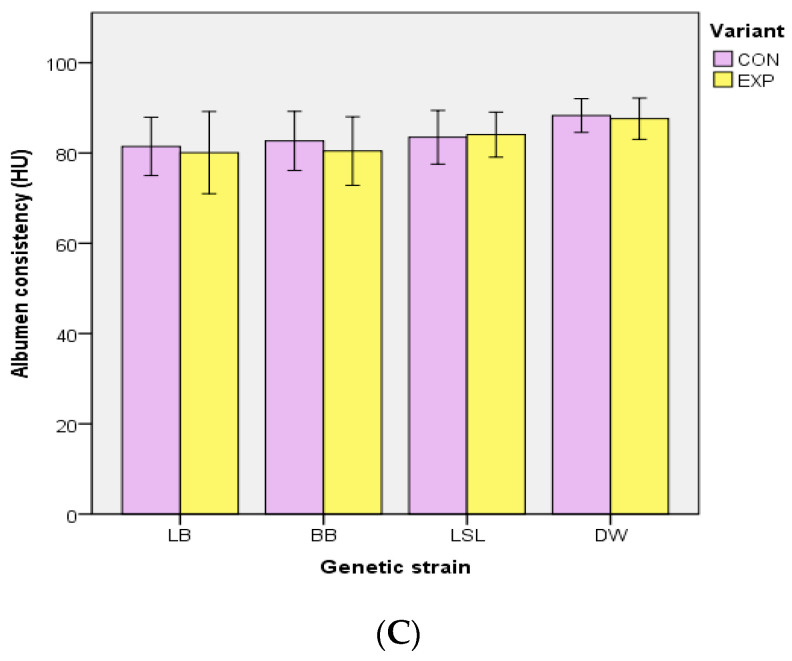
Effect of edible enrichment materials on albumen consistency in different layer strains in weeks 42 (**A**), 58 (**B**), and 67 (**C**). In a repeated-measures ANOVA model, lower HU values were found in the eggs of LB (*p* < 0.001), BB (*p* < 0.001), and DW (*p* = 0.027) within EXP compared to CON, but not in LSL (*p* = 0.642). HU–Haugh Units; CON-control group (without additional enrichment materials) EXP—experimental group (supply of additional enrichment materials); LB = Lohmann Brown classic; BB = Bovans Brown; LSL = Lohmann Selected Leghorn classic; DW = Dekalb White. Error bars represent standard deviation.

EM supply had no effect on the breaking strength of the egg shells (week 42—CON: 45.4 ± 8.8 N, EXP: 45.1 ± 8.8 N; week 58—CON: 41.7 ± 8.8 N, EXP: 41.0 ± 8.5 N; week 67—CON: 39.7 ± 9.0 N, EXP: 39.6 ± 9.1 N) (*p* = 0.765). In contrast, access to EM reduced the albumen consistency (*p* < 0.001), with a significant interaction between the strain and group (*p* = 0.033). The albumen consistency was 88.5 ± 5.4 HU (CON) and 87.4 ± 5.4 HU (EXP) in week 42, 85.4 ± 5.8 HU (CON) and 84.1 ± 6.5 HU (EXP) in week 58, and 84.0 ± 6.3 HU (CON) and 83.0 ± 7.4 HU (EXP) in week 67, respectively. Compared to CON, lower HU values were found in the eggs of LB (*p* < 0.001), BB (*p* < 0.001), and DW (*p* = 0.027) within the EXP, but not in the LSL (*p* = 0.642) (Figure 5).

## 4. Discussions

This study investigated the effect of an additional EM supply in littered housing systems on the integument condition and performance of different layer strains. The obtained results regarding integument condition traits provide new insight into several aspects. The positive effects of EM on plumage condition over all strains confirm the results of previous studies [6,26,27,28,29,30,31]. At the same time, the results are contrary to studies that did not observe such advantages of an EM supply [16,32,33,34]. Thus far, as the main reason for the inconsistent results between the studies, the different EM materials and their characteristics have been discussed [31]. Furthermore, study-associated differences in the housing and/or management conditions may have triggered these differences. However, the GEI results regarding EM effects revealed in our study indicate that the strains may also contribute to the differing findings. Possible causes for the overall reduction in plumage damage in the groups with pecking stones and alfalfa blocks could include (1) the promotion of foraging and feed intake behavior, which is considered a central factor in avoiding feather pecking [2,5,59]; (2) the fact that the use of pecking stones has an abrasive effect on the beak horn [31], making the protruding beak tip less effective for pecking feathers [60]; and (3) that additional nutrients/active substances ingested with EM reduce the risk of behavioral disorders at the animal nutritional level, as is known to be the case for sodium and crude fibre [61,62].

The EM supply was able to reduce skin lesions in LSL in week 72 but increased the prevalence in BB in week 43. Logistic regression models revealed significant interactions between strain and EM group for skin injuries as well as for plumage damage, which indicated the presence of GEI in these integument traits. Regarding plumage condition, EM had a positive effect in LB and LSL but a negative effect in BB. EXP groups in DW exhibited higher plumage damage in week 43 but lower damage in week 72 in comparison to CON. GEIs outlined as significant interactions between the genetic strain and the housing system were observed by [36,37,38,63,64,65] for various performance characteristics, including feed consumption and egg quality. Additionally, the GEIs between layer strains and housing systems for mortality were demonstrated within different brown egg-layer strains [66] and between white- and brown egg layers [67]. With the exception of the study by [31], the identification of GEIs in the supply of EM in littered housing systems has not yet been reported, because the majority of previous studies investigated only a single strain [6,16,26,27,28,29,30,32,33,34]. In furnished cages that were enriched with sand or sawdust, behavior and integument-associated interactions were shown by [68]. In Hy-Line White and Hy-Line Brown hens, an interaction between genotype and litter was found for the traits of body mass, claw length, and heterophilic/lymphocyte ratio as a stress indicator, but not in plumage condition and mortality. [69] observed no interaction between the group size and plumage condition of laying hen parent-stock flocks. Moreover, GEIs for the nesting behavior of laying hens are also known [70]. In turkeys, [71] found divergent effects between the sexes in cannibalism-induced animal losses when EM was used. The animal losses due to cannibalism in male turkeys were significantly higher in the groups with EM compared to the control groups, whereas EM had no statistically significant effect on mortality in turkey hens.

Summarizing the results of previous studies, interactions between genotypes and the environment have already been identified in individual behavioral or integument condition traits. With our study, these interactions were extended for an EM supply regarding plumage damage and skin injuries. Differences in the expression of genes relevant for feather pecking could be potential causes of this [72]. Furthermore, the divergent effect in the corticosterone reaction patterns between different laying hen strains after an additional intake of fibrous substrates could also offer an explanation [73]. However, the previously feared suppression of compound feed intake by EM [74] is not a main cause in the present study, as there were no significant differences in daily feed consumption between CON and EXP. The divergent response of the strains to EM access concerning the plumage condition indicates that—in the multifactorial causal structure of feather pecking—the weighting of the individual risk factors may differ between the strains, which should be verified by further studies.

Cannibalism is an important cause of animal loss in laying hens [49], with a high positive correlation between plumage and/or skin damage and the occurrence of cannibalism losses [75]. A significantly higher plumage damage and numerically higher animal losses due to cannibalism were also observed in the LB CON groups in our study. However, significantly lower plumage and skin damage with numerically lower cannibalism losses were observed in the BB CON groups compared to EXP. This underlines the relevance of the observed GEIs. The high animal losses (>10%) in 3 of the 24 compartments were mainly due to cannibalism of the skin (LB) and toes (LSL). In the view of the wide range between minimum and maximum in animal losses, the numbers of compartments per variant and runs should be increased in future studies to identify possible effects of EM in that trait.

Toe injuries are indirect indicators of toe pecking, which is a serious problem for animal health and welfare [12]. As in our study, previous investigations found toe injuries primarily in white egg layers [4,49,76]. Molecular genetic investigations confirmed their genetic predisposition for toe pecking [11]. In a recent laying hen performance test, however, toe injuries were also identified in DW [49]. For the first time, an increase in the prevalence of toe injuries with an EM supply could be shown in the present study, although final explanations are lacking. However, since the offer of EMs is accompanied by changes in the frequency of the use of certain stable areas [32], we cannot exclude the possibility that the more frequent flying arrival and departure events from equipment elements in EXP increased the risk of technopathic toe injuries. Subsequently, pre-existing toe injuries may trigger toe pecking [11]. Since toe pecking can cause death [77], the numerically higher animal losses due to toe injuries in the LSL EXP groups (3.8% vs. 0.9%) seem remarkable.

Hens reared with EM had lower body weights in weeks 8 and 16 compared to the CON, as already shown by [78]. Neither laying performance nor egg mass production increased with access to EM, as confirmed by recent studies [16,30,33,78]. In contrast to this study, [78] found higher egg weights in groups with access to pecking stones and alfalfa bales during the laying period. To our knowledge, no effect of EM provision in littered housing systems on albumen consistency as a trait of internal egg quality has been reported yet. CON eggs showed higher HU values in comparison to EXP. Other studies that examined this context found no unidirectional effect of the EM variant [28,78]. Possible nutritional causes for the reduced albumen consistency when EMs are ingested can be discussed. Elsherif et al. [79] found a reduced albumen consistency with the additional intake of copper in compound feed. In our study, pecking stones contained 90 mg/kg copper, which could explain the observed effect. Furthermore, a significant interaction between strain and EM group was observed for albumen consistency. With the performed study design, it was not possible to explain why EM effects in DW were not analogous to the three other strains. A GEI regarding albumen consistency was also identified by [68] when comparing the white egg-laying strain LSL with the brown egg-laying strain Atak-S kept in cage systems or with organic free-range farming, respectively. These differences in albumen consistency might be of interest for questions concerning the marketing of table eggs.

Overall, an improvement in the plumage and skin condition of laying hens can be achieved by providing additional EM in littered housing systems. However, since the effect of EM on integument condition differs between the strains, an opposite effect may also occur in certain hybrid lines. The identified GEI in integument traits should be investigated further in additional laying hen strains. Moreover, it is recommended to include as many different strains as possible and an increased number of repeats (i.e., compartments per strain and group and runs) in future studies on the effects of EM in pullets and laying hens. Due to distinct differences in integument conditions between the strains and the interaction with the housing environment, a scoring for plumage, skin, and toe condition should be implemented in laying hen performance tests. Finally, in the view of the divergent effects of EM between the strains, practical recommendations for the supply of EM should be strain-specific. In LSL hens, the provision of EM must be considered carefully on a herd-specific basis, since, although improvements in plumage and skin condition can be expected, toe injuries can occur more frequently.

## Figures and Tables

**Table 1:** Effect of strain, age, enrichment group, and interaction strain * enrichment group on integument condition—results of the logistic regression models.

Trait	Score 1 (%)	Coefficients (SE)	Odds Ratio (95% CI)	Individual *p*-Value	Overall *p*-Value
**Total Plumage Score**					
LB	45.6	reference	baseline		<0.001
BB	52.7	−0.74 (0.31)	0.48 (0.26–0.87)	0.016	
LSL	46.4	0.14 (0.31)	1.14 (0.63–2.09)	0.662	
DW	35.6	−1.59 (0.32)	0.20 (0.11–0.38)	<0.001	
week 20	1.3	reference	baseline		<0.001
week 43	40.7	4.35 (0.38)	77.43 (36.47–164.36)	<0.001	
week 72	93.2	7.69 (0.42)	2199.89 (963.13–5024.78)	<0.001	
CON	45.2	reference	baseline		0.001
EXP	44.9	−1.09 (0.32)	0.34 (0.18–0.63)		
compartment	-	0.04 (0.01)	1.04 (1.01–1.06)		0.003
CON × LB	49.8	reference	baseline		<0.001
EXP × BB	61.3	3.24 (0.49)	25.49 (9.79–66.20)	<0.001	
EXP × LSL	40.4	−0.09 (0.44)	0.91 (0.38–2.17)	0.838	
EXP × DW	36.4	1.22 (0.44)	3.40 (1.43–8.09)	0.006	
constant		−4.66 (0.45)			
**Skin Injuries**					
LB	11.6	reference	baseline		0.632
BB	15.3	−0.19 (0.32)	0.83 (0.44–1.56)	0.560	
LSL	10.0	0.11 (0.31)	1.11 (0.61–2.06)	0.728	
DW	8.9	−0.26 (0.32)	0.77 (0.41–1.45)	0.416	
week 20	0.0	reference	baseline		<0.001
week 43	5.8	18.41 (1620.70)	50.71 (0.74–77.51)	0.991	
week 72	28.5	20.32 (1620.70)	1008 (0.89–2171.81)	0.990	
CON	11.9	reference	baseline		0.436
EXP	11.0	−0.26 (0.33)	0.77 (0.40–1.48)		
compartment	-	0.01 (0.01)	1.01 (0.98–1.04)		0.527
CON × LB	12.4	reference	baseline		0.001
EXP × BB	19.6	1.14 (0.47)	3.14 (1.24–7.91)	0.016	
EXP × LSL	6.2	−0.74 (0.49)	0.48 (0.18–1.26)	0.134	
EXP × DW	7.6	−0.13 (0.48)	0.87 (0.34–2.26)	0.784	
constant		−21.20 (1620.44)			

interaction strain * enrichment group – statistical interaction between strain and enrichment group, SE—standard error; CI—confidence interval; LB—Lohmann Brown classic; BB—Bovans Brown, LSL—Lohmann Selected Leghorn classic; DW—Dekalb White; CON—control group (without additional enrichment materials); EXP—experimental group (supply of additional enrichment materials); Score 0—intact plumage/skin; Score 1—plumage/skin damage.
